# Deciphering the Molecular Signatures and Optical Properties of Dissolved Organic Matter in the Aksu River (Xinjiang, China) via FT-ICR MS and 3D-EEM Spectroscopy

**DOI:** 10.3390/ijms27052246

**Published:** 2026-02-27

**Authors:** Fengjun Shao, Alimir Ablikumu, Yimo Wang

**Affiliations:** 1 Key Laboratory of Green and Efficient Mining and Ecological Restoration in High-Altitude Arid Regions of Xinjiang, Urumqi 830047, China; 2 School of Geology and Mining Engineering, Xinjiang University, Urumqi 830047, China; 20222705817@stu.xju.edu.cn (A.A.); prjwym18133893960@163.com (Y.W.)

**Keywords:** dissolved organic matter (DOM), optical properties, molecular characteristics, FT-ICR MS, desert oasis river system

## Abstract

Desert oases and river systems are complex and dynamic ecosystems featuring unique hydrological patterns. The system significantly influences the production, degradation, and transformation of dissolved organic matter (DOM), thereby further regulating DOM in the desert oasis. However, the molecular composition and significance of DOM in rivers within desert oases are rarely studied. In this paper, the optical properties and spatial variation in molecular characteristics of surface water DOM in the Aksu River were investigated using three-dimensional fluorescence spectroscopy (3D-EEM) and Fourier transform ion cyclotron resonance mass spectrometry (FT-ICR MS). The results indicated that the rivers possess distinct molecular compositional characteristics of DOM, with high spatial heterogeneity (variations in optical parameters and molecular compounds). Diversity in DOM is revealed at the molecular level primarily through S- and N-containing functional groups. Unlike large rivers (e.g., the Yangtze) dominated by terrestrial inputs or algal blooms, our study reveals that the DOM in the Aksu River (a desert-oasis river) is characterized by highly unsaturated and phenolic compounds and is primarily driven by intense photodegradation and evaporation rather than by microbial or terrestrial allochthonous inputs. This highlights a distinct photochemical signature unique to arid river systems. The findings will deepen the understanding of the DOM in desert-oasis river systems. Based on this research, seasonal variation in DOM in the Aksu River under different hydrological conditions can be further studied, thereby enriching the understanding of the carbon cycle in desert-oasis river systems.

## 1. Introduction

Dissolved organic matter (DOM), an essential component of water quality, is ubiquitously present in natural environments, including soils, sediments, lakes and rivers, seawater, and aerosols [1,2,3,4]. As the primary form of organic carbon in aquatic systems, DOM plays a role in many biogeochemical processes such as the global carbon cycle, the transport of nutrients, and pollutants [5,6,7,8]. It has significant impacts on the global carbon and nitrogen cycles, climate change, and human health. In inland waters, the primary sources of DOM are local (aquatic plants, phytoplankton, and sediments) and external (terrestrial material inputs) [9,10]. The diversified biogeochemical processes and origins can result in complex and heterogeneous DOM, which have profound effects on the environment [1,5]. Exploring the composition and characteristics of DOM is crucial, as it offers insights into its sources, biogeochemical processes, environmental behaviors, and its role in aquatic systems.

DOM is a heterogeneously complex organic compound produced by various abiotic and biotic transformations, characterized by different molecular sizes, structures, and functional properties. DOM molecules are among the most common components in aquatic and terrestrial ecosystems, primarily composed of carbon, hydrogen, and oxygen, with smaller contributions from non-oxygen heteroatoms such as nitrogen, sulfur, and phosphorus [11,12,13].

In recent years, with the rapid development of mass spectrometry technology, Fourier Transform Ion Cyclotron Resonance Mass Spectrometry (FT-ICR-MS) has been widely used for the chemical and molecular composition characterization of DOM [14,15,16]. Combined with spectroscopy techniques (such as UV-visible absorption spectroscopy or three-dimensional fluorescence spectroscopy), the fundamental composition and sources of DOM can be identified by tracking fluorescent chromophores. This provides a more comprehensive insight into the origins and molecular characteristics of DOM from various aquatic systems. For instance, terrestrial indicators of optical parameters (like the humification index, SUVA254) are closely related to compounds identified by FT-ICR MS (polyphenols and highly unsaturated compounds), indicating terrestrial inputs of DOM [8,17].

Existing research suggests that the chemical properties of freshwater DOM are closely associated with numerous environmental factors (such as water sources, hydrology, climate, etc.) and human activities [8,18,19,20]. For example, there are significant differences in the spatial distribution of DOM in inland lakes and rivers. Research on DOM in Taihu (China) indicates that its composition and characteristics are highly consistent, whereas the molecular composition of river DOM displays significant spatial variations [19]. Specifically, the quantity and relative intensity of sulfur-containing compounds continuously increase from upstream to downstream. Human activities, such as wastewater discharge or ship transportation, might also introduce additional organic substances (like linear alkylbenzene sulfonate) into aquatic systems, emphasizing the impact of anthropogenic sources [21].

Research characterizing DOM at the water molecule level in rivers within desert oases is very limited, which restricts our ability to identify the sources and processing pathways of DOM in such water bodies. The composition of water body DOM affects water quality parameters driven by DOM degradation (such as dissolved oxygen), which may affect human health. Additionally, river water DOM has been shown to drive the food webs of stream ecosystems [22,23].

The molecular characteristics of DOM vary depending on its origin, which in turn influences its bioavailability and reactivity [1,13]. While there is increasing understanding of the characteristics of DOM in riverine and marine envir`onments, the sources, composition, characteristics, and ultimate destination of DOM in rivers within desert oases remain unclear. Therefore, analyzing the chemical composition of DOM in desert-oasis river systems at the molecular level can offer better insights into the roles of DOM in biogeochemical reactions, global carbon cycling, heavy metal transport, water treatability, and potability.

The main objectives of this study are: (1) Employing three-dimensional fluorescence spectroscopy in tandem with the fluorescence regional integration method, our aim is to delineate the primary sources and composition of DOM in the surface water of the Aksu River basin. (2) Utilizing negative ion electrospray ionization Fourier transform ion cyclotron resonance mass spectrometry, we aim to uncover the chemical, physical, and/or biological transformations in DOM as it transits from upstream to downstream, and to suggest potential underlying mechanisms. It is expected that this study will enhance understanding of the DOM in desert-oasis river systems. In addition, this work will drive further research on seasonal variations in DOM under different hydrological conditions in desert-oasis river systems, thereby enriching our knowledge of the carbon cycle in these systems.

## 2. Results and Discussion

### 2.1. Optical Properties of DOM

The FI value is commonly used to identify the source of the DOM type of humic substances. When FI > 1.8, it indicates that the main source of DOM is from bacterial and algal activities, belonging to endogenous production (the autogenic characteristics of DOM are more obvious). When FI < 1.2, it indicates that DOM originates from terrestrial plants and soil organic matter, belonging to exogenous (terrestrial) input (the allochthonous characteristics of DOM are more obvious). When 1.2 < FI < 1.8, it indicates that DOM is produced both endogenously and exogenously [24,25]. The range for samples S1–S9 is 1.61–1.80 (with an average value of 1.70) (Table 1). Clearly, the FI values of samples from the study area are between 1.2 and 1.8. This suggests that the DOM in the Aksu River could originate from both exogenous (terrestrial) input and endogenous production from microbial activities. Several recent studies have shown that in most natural aquatic systems, the FI value is typically between 1.2 and 1.8 [26,27], which is consistent with the results of this paper.

The BIX value represents the contribution of autotrophic productivity to DOM (Dissolved Organic Matter). A higher value (>1) indicates an autogenic source resulting from biological or bacterial activity, indicating a predominance of fresh endogenous organic matter. In contrast, a lower value (<0.6) indicates a strong exogenous organic matter signature, implying significant anthropogenic influence [28]. The BIX values for samples S1–S9 range between 0.46 and 1.60 (with an average of 0.80) (Table 1). Most samples have BIX values between 0.6 and 1.0, suggesting that the DOM has moderate autogenic characteristics, consistent with microbial metabolism and terrestrial input. At the same time, some sampling points, such as S6, show apparent human activity influence. This is reasonable given the various human activities (such as agriculture, fishing, and livestock breeding) that frequently occur along the riverbanks. As one progresses downstream, human activities become increasingly rich and diverse.

The HIX value characterizes the degree of humification of DOM [29]. A high HIX indicates greater humification, suggesting that the DOM is more stable. The HIX values for samples S1–S9 in the study area range from 0.66 to 1.00, with an average value of 0.91 (Table 1). This suggests that the degree of humification of DOM in the water bodies within the study area is relatively low, and that DOM stability is suboptimal, making it susceptible to external inputs.

**Figure 1:**
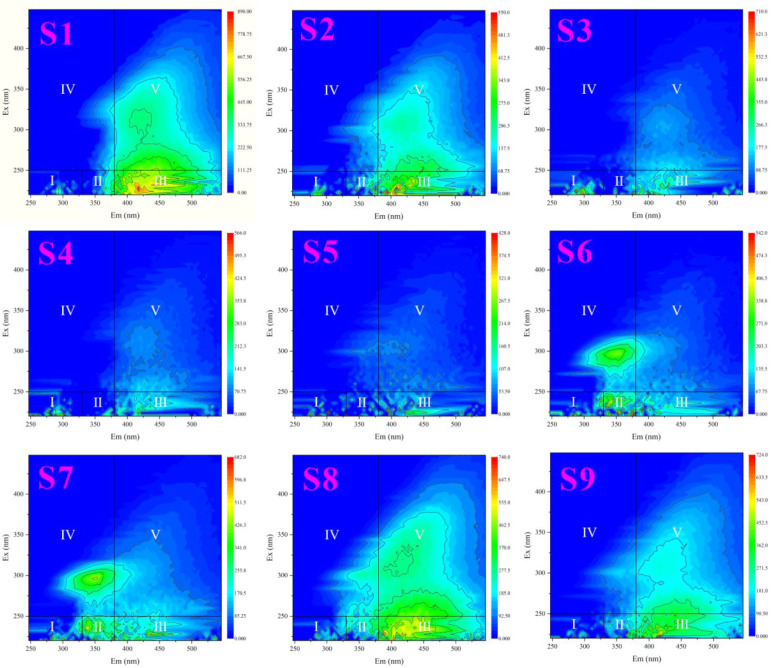
Characteristics and distributions of EEMs spectra categorized by the specified five regions in FRI analysis for S1−S9.

**Figure 2:**
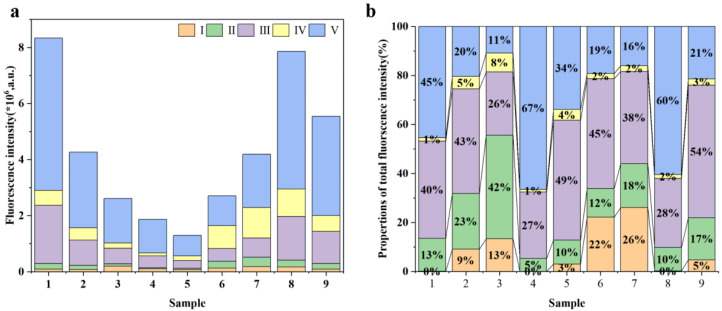
Fluorescence intensity (**a**) and relative proportion (**b**) of each FRI region for samples S1–S9.

In the FRI analysis, the EEM spectra of all samples are divided into five regions (Table 2) [30]. Regions I (tyrosine-like proteins), II (tryptophan-like proteins), and IV (Microbial-like) are classified as protein-like substances, possibly related to the products of microbial transformations. Regions III (Fulvic acid-like) and V (humic-like substances) are considered humic-type substances, possibly associated with terrestrial input [31,32]. There is a clear spatial heterogeneity among the samples, with the relative proportions in each region being distinct (Figure 1 and Figure 2). Among them, Fulvic acid-like (25.72–54.01%) and humic-like substances (10.88–66.55%) are the dominant components. The spatial heterogeneity among samples is speculated to be related to the complex geological environment of the study area, seasonal flood confluences, and diverse human activities along the banks (such as agricultural production, livestock breeding, and urban life).

For sample S1, the humic-like substances (45.36%) and Fulvic acid-like (39.68%) components were the most abundant (Figure 2). Previous studies reported that rivers with strong DOM were observed to have a higher proportion of humic-like and fulvic-like substance components. As mentioned earlier, the sampling site for sample S1 is located at Duolang Lake in the downstream of the Aksu River. Compared to other sampling points, it has a more stable hydrological environment. Meanwhile, human activities are more concentrated around it, leading to a wider range of material sources. This likely results in the pronounced presence of humic-like and fulvic-like substance components in the samples from this location.

### 2.2. Molecular Diversity of DOM Analyzed by FT-ICR MS

To investigate the molecular differences in DOM in the upstream and downstream river waters, S9 from the upstream and S2 from the downstream were selected for FT-ICR MS mass spectrometry analysis.

**Figure 3:**
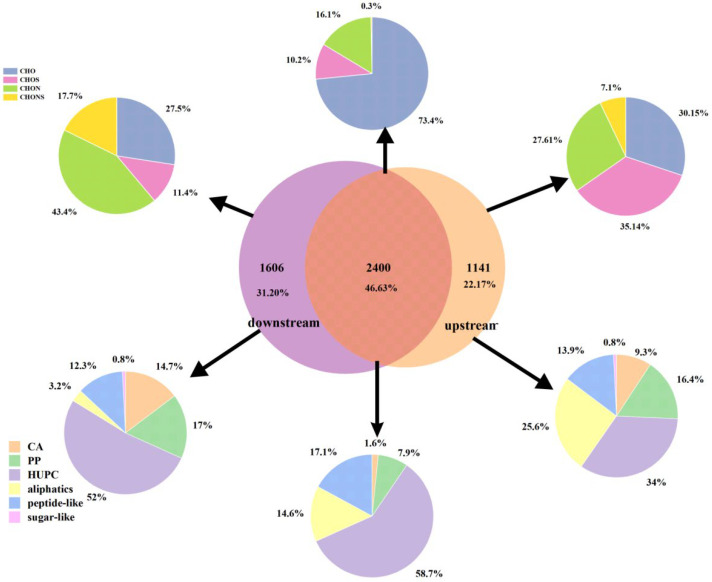
The Venn diagram for the comparison of the unique and overlapping assigned molecular compounds in the downstream and upstream samples.

**Figure 4:**
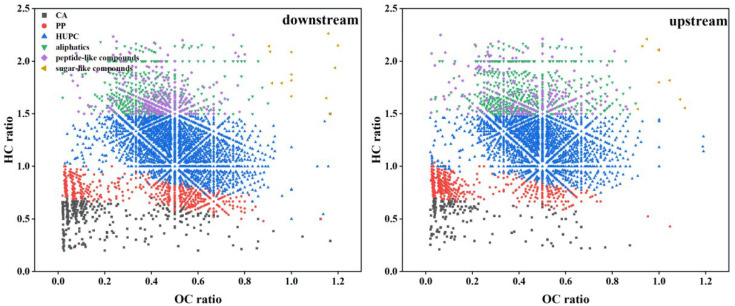
Van Krevelen diagrams on different classes of compounds of downstream and upstream samples.

The peak distribution of the FT-ICR MS spectra in DOM samples is similar, with thousands of peaks identified in the 100–800 m/z range. However, based on the extended standard mass, differences in components and structures can still be observed, as well as unique molecular formulas identified in each sample. This result underscores the powerful advantage of FT-ICR-MS in characterizing complex DOM compounds. A total of 5147 molecular formulas were identified in the downstream (S2) and upstream (S9) samples, including 2400 common formulas/shared formulas (appearing in all samples) and 2747 unique formulas (sum of formulas only appearing in each sample) (Figure 3). Interestingly, CHO formulas dominate in the common formulas (53.23% upstream, 49.48% downstream), while a higher proportion of heteroatom formulas (CHOS, CHON, and CHONS) were found in unique formulas. These results suggest that the Aksu River samples have high chemical diversity in DOM, primarily driven by heteroatom compounds. This high diversity is also evident in the van Krevelen (v-K) diagram (Figure 4), where unique formulas are widely distributed across the entire v-K diagram, while common formulas are more clustered.

CHO and CHON are the most abundant molecular formulas in each sample, with molecular weights ranging between 1885–1982 and 841–1222, respectively, followed by CHOS (506–724) and CHONS (92–295).

Based on the identified molecular formulas, various compound groups were classified to determine the DOM composition and sources (Figure 4). Condensed aromatic compounds (CA) originate from the combustion of biomass and fossil fuels or from humification processes within terrestrial systems without combustion [33,34]. Upstream levels are slightly lower (4.29%) than downstream (7.06%). This aligns with reality, where upstream mountain areas are more developed, while flat downstream areas are more conducive to human habitation and production. This further indicates that the scouring effects of upstream water can bring more terrestrial organic matter (such as soil) into the downstream basin, leading to a higher proportion of polycyclic aromatic compounds downstream. However, in classic lakes, there is almost no spatial difference in CA [19,21], possibly because they lack significant spatial variation in hydrological patterns (flow rate, etc.). The upstream Aksu River (Toshkan River basin) is also rich in petroleum (oil and gas) resources, which could be a significant external factor.

Polyphenolic compounds (PP) typically come from vascular plants and are sensitive to photodegradation. They show a slight increasing trend from upstream to downstream, rising from 11.55% to 12.35%. This could be due to upstream-to-downstream vascular plant input and more abundant agricultural activity downstream than upstream. Additionally, the research area receives abundant sunlight and longer daylight hours, both of which contribute to downstream PP being higher than upstream.

There is a noticeable difference in highly unsaturated compounds (HUPCs) between upstream and downstream samples, with upstream HUPCs decreasing from 57.58% to 62.07% downstream. This could be due to the downstream riverside soil becoming more fertile, with increased agricultural production and algal organic matter, making downstream HUPC richer than upstream.

Moreover, some unsaturated aliphatic compounds were detected in DOM samples, which are usually derived from bacterial and algal metabolic products [34,35]. However, there is no decreasing trend for these compounds between the Aksu River’s upstream and downstream. This could be due to photodegradation of high aromatic substances (like polyphenols), which can transform into aliphatics (photo-products).

### 2.3. Molecular Composition/Chemical Evolution

FT-ICR MS identified 3541 and 4006 molecular formulas in the upstream (S9) and downstream (S2) samples, respectively. The average molecular formula for the upstream sample is C_21.19_H_26_O_8.56_N_0.38_S_0.23_ (average molecular weight 398.67), and for the downstream sample, it is C_21.52_H_24.51_O_9.30_N_0.55_S_0.20_ (average molecular weight 409.90).

The Hydrogen to Carbon ratio in the downstream sample is lower than in the upstream sample (H/C: 1.17 vs. 1.26). This indicates that compounds in the downstream sample have a higher degree of structural saturation. Based on calculations from all molecular formulas, the downstream sample indeed shows a higher AImod value (0.27 ± 0.01 vs. 0.22 ± 0.00) and DBE value (10.54 ± 0.10 vs. 9.39 ± 0.10) compared to the upstream sample. For instance, in Poyang Lake [20], the DOM is primarily influenced by hydrological connectivity and is rich in protein-like substances. In contrast, our data (S2 vs. S9) show a dominance of humic-like and highly unsaturated compounds (HUPC, 57.58–62.07%), suggesting that photochemical maturation is a more dominant process in the Aksu River than the hydrological connectivity effects observed in river-connected lakes [8].

**Figure 5:**
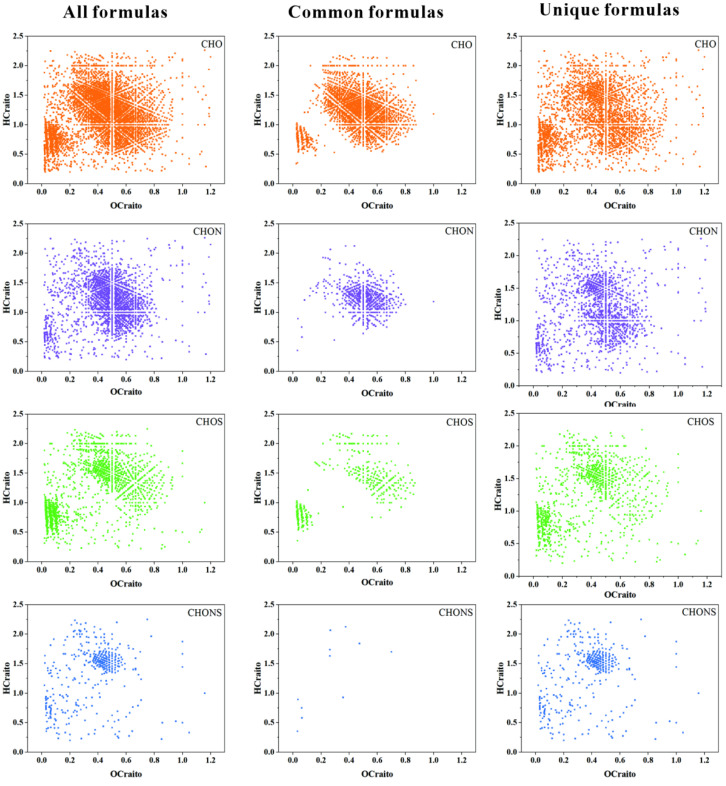
Van Krevelen diagram of all formulas, common formulas, and unique formulas in S2 and S9.

The comparison of the DOM molecular composition between these two types of samples is shown as a Venn diagram (Figure 3). All the upstream and downstream molecules are divided into three groups: unique to upstream or downstream, shared by both, and the total from both (Figure 5). Overall, overlapping molecules account for 46.63% of all molecules, indicating a high similarity between the two types of samples. The downstream sample displays a higher percentage of unique molecular compounds than the upstream sample (31.20% vs. 22.17%).

In the upstream (53.23%) and downstream (49.48%) samples, the proportion of CHO molecules is comparable. For CHON molecules, the upstream (23.75%) is noticeably lower than the downstream sample (30.50%). The high proportion of nitrogenous compounds aligns with the agricultural production along the river basin. The downstream region has more advanced agriculture than the upstream, suggesting that the introduction of nitrogen sources during agricultural production might be a major influencing factor.

In large rivers (e.g., Poyang Lake), sulfur-containing components may result from pollution. However, in the Aksu River, they may be related to sulfurization reactions in arid areas or to specific mineral interactions, processes unique to this environment. Sulfur-containing molecules (CHOS and CHONS) in the upstream sample (23.04%) are more enriched than those in the downstream sample (20.00%). Apart from contributions from streams, rainfall, and the melting of ice and snow in upstream areas, the higher sulfur content in the upstream might also be attributed to sulfidation reactions. At a particular pH, thiol groups(R-SH) may be involved in the formation of more stable organic-mineral complexes. Sulfates (SO_4_^2−^) bind to highly reduced organics in the form of thiols (R-SH) or thioethers (R-S-R’) [36].

The van Krevelen diagram is used for graphical analysis, complementing the plotting of elemental ratios (O/C and H/C) for complex DOM compounds, and can identify and depict changes in sources and overall DOM composition (Figure 4).

The dominant compounds in the downstream sample are highly unsaturated and phenolic compounds (HUPC) (62.07%) and polyphenolic compounds (PP) (12.35%). In contrast, the predominant compounds in the upstream sample are highly unsaturated and phenolic compounds (HUPC) (57.58%) and aliphatic compounds (19.85%) (Figure 4). The downstream sample has a higher contribution of unsaturated compounds than the upstream sample. Aliphatic compounds (more upstream) are often susceptible to microbial mineralization into CO_2_ [37,38,39,40]. However, if divalent cations (such as Ca^2+^, Mg^2+^, which are abundant in arid waters) are present, they can act as a “bridge” connecting negatively charged DOM to negatively charged minerals.

In both upstream and downstream samples of the river basin, HUPC remains the most significant compound. Previous studies suggest that photochemically produced intermediates containing singlet oxygen (1O2) and hydroxyl radicals (•OH) might preferentially react with nucleophiles and oxidize aromatic rings, resulting in more oxidized products. Both oxidation processes correspond to the observed elevation in the O/C ratio. Aromatic (CA) and Highly Unsaturated (HUPC) compounds, due to their stable structure, are more likely to be converted to Mineral-Associated Organic Matter (MAOM) or early diagenetic products preserved in sediments. In the samples, components shift from aliphatic and peptidic natures to more oxidized HUPC and PP compounds [41,42,43]. Moreover, compared with the upstream sample, the downstream sample shows a more pronounced aromatic character (7.06% vs. 4.29%).

While flood pulses in the Yangtze River [20] introduce diverse terrestrial molecules, the increased molecular diversity (specifically CHON and CHOS) observed downstream in the Aksu River is likely attributed to agricultural runoff (higher CHON) and photochemical sulfurization under high evaporation, rather than simple terrestrial runoff. Using FT-ICR MS molecular analysis, we can determine that the spatial heterogeneity in molecules between upstream and downstream samples in the Aksu River basin is primarily due to a combination of microbial activity, photochemical reactions, and human activities (such as agricultural production and livestock farming).

### 2.4. Impact on Regional Carbon Cycle and Further Considerations

This study provides a comprehensive spectroscopic and molecular-level analysis of the origin and characteristics of DOM in the desert-oasis inland river, the Aksu River. The DOM of desert-oasis inland rivers exhibits distinct spatial heterogeneity, with the unique features of the Aksu River’s DOM implicating various biogeochemical processes. Since DOM composition is closely linked with the burial of organic carbon in freshwater systems and the release of CO_2_ and CH_4_, desert-oasis inland rivers might have different carbon cycle dynamics than conventional lakes and rivers, warranting special attention when studying carbon emission and sequestration in desert-oasis waters [8,44,45].

Furthermore, our research highlights the main spatial differences: DOM in the upstream has a higher proportion of refractory molecules, indicating better stability than downstream. As stable DOM is usually closely associated with the burial and preservation of organic matter in sediments, upstream DOM is more likely to be buried [8,46].

Serving as an inland arid-semiarid desert-oasis river with a unique hydrological pattern, the Aksu River may transport carbon downstream (into the Tarim River). In addition, it still receives and retains carbon from its vast catchment area, akin to lakes. It is speculated that carbon burial, emission, and transport fluxes in desert-oasis rivers are more complex than in standalone lakes or rivers, which is determined by their geological settings.

Additionally, influenced by its unique climate and topography, the Aksu River displays a seasonal hydrological pattern (four distinct periods: dry, rising, flood, and recession). The source of DOM might vary between these periods, e.g., more terrestrial organic input from surrounding wetland vegetation during the flood season than the dry season [47]. With the complex hydrology of the desert-oasis river system, DOM is perceived as being spatially and temporally dynamic, crucial for understanding river-connected carbon cycling.

While this research characterized the source, composition, and distribution of DOM in the Aksu River, some limitations persist. Firstly, seasonal variations in the Aksu River’s DOM were not considered; existing studies suggest significant changes in DOM molecular characteristics between dry and flood periods, complexation of iron/aluminum oxides or clay minerals with DOM is common in arid rivers due to flow rate variation and intense evaporation [48]. Secondly, this study primarily focuses on the main channel of the Aksu River, while surrounding land uses (saline soils, grasslands, deserts, farmlands, urban areas, etc.) and their impact on DOM remain unexplored. Therefore, further research in these areas is essential to better understand the biogeochemical processes of the Aksu River’s DOM, enriching our knowledge of carbon cycling in desert-oasis river systems.

## 3. Materials and Methods

### 3.1. Sites and Sampling

#### 3.1.1. River Description

**Figure 6:**
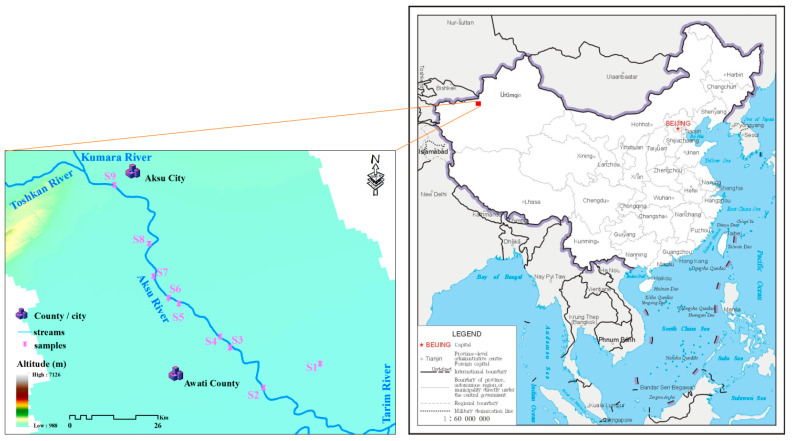
Map of the sampling sites in the Aksu River.

The Aksu River is located in the Aksu region of northwest Xinjiang, China, on the northern edge of the Tarim Basin. It is a typical river within a desert oasis. Formed by the confluence of the Toshkan River (from the west) and the Kumalak River (from the north), after the confluence, it flows southwards and empties into the Tarim River (Figure 6) [49,50].

The Aksu River is 224 km long, and its waters are mainly replenished by mountain precipitation and the melting of ice and snow [50]. The basin has a temperate continental climate, ranging from arid to semi-arid. The annual average precipitation is 40.0–50.0 mm [51], with the north generally receiving more precipitation than the south. The average annual temperature ranges from 9.1∼10.7 °C. The terrain slopes from northwest to southeast. The northwestern part of the river is bordered by the La Ta Jia Yi mountains (part of the Tianshan range), which are oriented northwestward, with the highest point at 2759 m. The southern part of the river lies in a typical alluvial plain at an altitude of 1100 m. The average slope in the plain area is between 1‰ and 3‰ [49,50,51,52].

#### 3.1.2. Sample Collections and Procedure

Sampling was carried out in February 2023. In this sampling, nine surface water samples were collected. These included upstream samples (S9), downstream confluence samples (S2), and samples from Duolang Lake (S1), with the rest collected along the main river channel of the basin to study the DOM of the Aksu River (Figure 6).

All samples were obtained about 0.1m below the river’s surface and were stored in hydrochloric acid-cleaned Nalgene bottles. They were kept in a dark environment and were transported to the laboratory for relevant experiments within 7 days.

### 3.2. Measurement of DOC Concentrations

The DOC analyzer was used to measure DOC concentration in 10 mL of each filtrate. The acidified samples (with 100 μL of 10% hydrochloric acid added to every 10 mL sample) were combusted at 850 °C using O_2_ as the carrier gas. The carbon dioxide produced from combustion was detected through a non-dispersive infrared analyzer. The system was calibrated using a mixture of standards, including potassium hydrogen phthalate, sodium nitrate, and ammonium chloride.

### 3.3. D-EEM Fluorescence Measurements and Fluorescence Regional Integration

All samples were analyzed for three-dimensional excitation-emission matrix (3D-EEM) fluorescence at room temperature using a HORIBA Aqualog-UV-NIR-800 (Made in USA). The excitation source was a 700 V xenon arc lamp. Fluorescence was scanned with excitation wavelengths from 200 to 450 nm in increments of 5 nm and emission wavelengths from 200 to 450 nm in increments of 1 nm. Milli-Q water (18.2MΩcm−1) was used as a reference for quality control during testing. The Milli-Q water spectrum was subtracted from the obtained EEMs to minimize background interference. The EEMs were eventually normalized to Raman units (R.U.). The experiments were conducted in the laboratory of the School of Environment and Spatial Informatics, China University of Mining and Technology.

Optical parameters included the fluorescence index (FI), indicative of humic-like origin in DOM, the biological index (BIX), indicating primary biological activity and the source of DOM, and the humification index (HIX) representing the humification degree of DOM [29,53,54], along with fluorescence regional integration (FRI). FRI is a quantitative technique indicating specific fluorescent substances in DOM, dividing each EEM spectrum into five regions with continuous Ex and Em boundaries. A detailed description of this method has been reported in the literature [30,55].

### 3.4. Solid Phase Extraction of DOM and FT-ICR MS Analysis

Each DOM sample of 50 mL was acidified to pH 2 with formic acid, then extracted using an Agilent Bond Elute PPL cartridge (500 mg, 6 mL) via solid-phase extraction (SPE). The SPE DOM (SPEDOM) was analyzed in negative ion mode on a 15T Bruker Solari X FT-ICR MS equipped with an electrospray ionization source. The entire measurement procedure for ESI FT-ICR MS analysis followed previously published protocols [30,55,56]. After calibration using a reference molecular formula mass table, a mass error of less than 1 ppm was ensured for singly charged molecules over the entire mass range.

Molecular formula assignments were obtained from in-house software. Formula assignments were constrained to ^12^C_1−60_, ^1^H_1−120_, ^16^O_0−30_, ^14^N_0−3_ and ^32^S_0−1_, while isotopes ^18^O,^13^C, and ^34^S were not considered. Generated formulas were validated by setting reasonable chemical constraints [O/C ≤ 1, H/C ≤ 2_n + 2*_(C_n_H_2n + 2_)]. The number of nitrogen and sulfur atoms was restricted to three, as no mass distributions with more than three N and S atoms were observed in previous natural organic matter (NOM) studies [57,58].

FT-ICR-MS parameters, such as molecular formulas (CHO, CHOS, CHON, and CHONS), atomic ratios (i.e., H/C, O/C), double bond equivalents (DBE), and modified aromaticity index (AImod) were calculated through relative peak intensities. The peak values were obtained by dividing the size of each molecule by the total size of all designated molecular formulas in each sample. Calculations for DBE and AImod are detailed in Formulas (1) and (2).

Furthermore, assigned chemical formulas were divided into the following compound classes: condensed aromatics (CA/AI mod ≥ 0.67), polyphenolic compounds (PP: 0.67 > AI mod > 0.50), highly unsaturated and phenolic compounds (HUPC/AI mod ≤ 0.50, H/C < 1.5), aliphatic compounds (H/C ≥ 1.5, O/C ≤ 0.9, N = 0), peptide-like compounds (H/C ≥ 1.5, O/C ≤ 0.9, N > 0), and sugar-like compounds (H/C ≥ 1.5, O/C > 0.9) [56,57,58,59].

(1) DBE=1+0.5∗(2∗C−H+N)

(2) $$AI_{\mathrm{mod}}=\frac{1+C-\frac{1}{2}*O-S-\frac{1}{2}(N+H)}{C-\frac{1}{2}*O-N-S}$$

## 4. Conclusions

The study explored how the optical properties and molecular characteristics of surface water DOM in the Aksu River vary spatially. The desert-oasis river system, Aksu River, possesses unique DOM optical properties and molecular features, characterized by high spatial heterogeneity (variations in optical parameters and molecular compounds). Specifically, Highly Unsaturated and Phenolic Compounds (HUPC) were identified as the dominant fraction, increasing from 57.58% upstream to 62.07% downstream. Optically, the DOM exhibited mixed sources (FI: 1.61–1.80), but low humification (HIX < 1.0), distinguishing it from the highly humified DOM in terrestrial soils. The research highlights a unique nitrogen and sulfur-enriched molecular signature (CHON and CHOS), which accounted for a significant portion of the unique formulas (27.47% unique formulas), driven by the specific agricultural and evaporative conditions of the oasis.

The spatial heterogeneity in optical properties between upstream and downstream samples in the Aksu River basin is speculated to be related to the complex geological environment, seasonal flood inflows, and diverse human activities along the banks (agricultural production, livestock farming, and urban living). Meanwhile, the spatial heterogeneity of molecular compounds mainly results from the combined effects of microbial activity, photochemical reactions, and human activities (agricultural production, livestock farming, etc.).

## Figures and Tables

**Table 1:** Optical parameters of DOM samples based on EEM spectroscopy.

Sample	EEMs Parameters		
	FI	BIX	HIX
S1	1.68	0.81	0.99
S2	1.73	0.93	0.77
S3	1.69	1.60	0.66
S4	1.65	0.54	1.00
S5	1.71	0.75	0.96
S6	1.66	0.46	0.96
S7	1.61	0.60	0.89
S8	1.80	0.81	0.99
S9	1.79	0.69	0.96
Ave	1.70	0.80	0.91

**Table 2:** Descriptions of five regions by Fluorescence Regional Integration.

Region	Excitation (nm)	Emission (nm)	Subsantces
I	200−250	250−330	Tyrosine-like protein
II	200−250	330−380	Tryptophan-like protein
III	200−250	380−500	Fulvic acid-like
IV	250−280	250−380	Microbial-like
V	280−400	380−500	Humic-like

## Data Availability

All data generated or analyzed during this study are included in this published article.
